# MRI Findings in Acute Hyperammonemic Encephalopathy: Three Cases of Different Etiologies: Teaching Point: To recognize MRI findings in acute hyperammonemic encephalopathy

**DOI:** 10.5334/jbsr.2017

**Published:** 2020-01-30

**Authors:** Elisabeth Reis, Tim Coolen, Valentina Lolli

**Affiliations:** 1 Erasme Hospital, BE

**Keywords:** Acute hyperammonemic encephalopathy, magnetic resonance imaging (MRI), diffusion-weighted-imaging (DWI)

## Abstract

Acute hyperammonemic encephalopathy is a rare but life-threatening condition that might complicate liver disease as well as non-hepatic conditions. It can lead to coma and death, secondary to brain edema and intracranial hypertension. We present three cases of acute hyperammonemic encephalopathy of different etiologies and the observed brain MRI findings. Symmetrical extensive cortical signal abnormalities, typically involving the insular and cingulate cortices, often showing restricted diffusion, are commonly described. These specific imaging features should be recognized by the radiologist since prompt treatment of the condition is paramount.

## Introduction

Acute hyperammonemic encephalopathy is a serious condition that complicates chronic or acute liver disease, as well as non-hepatic conditions such as drugs or bacterial infections [1]. In this article, we present three cases of acute hyperammonemic encephalopathy of different etiologies and the associated brain MRI findings.

## Case Reports

### Case 1

**Figure 1:**
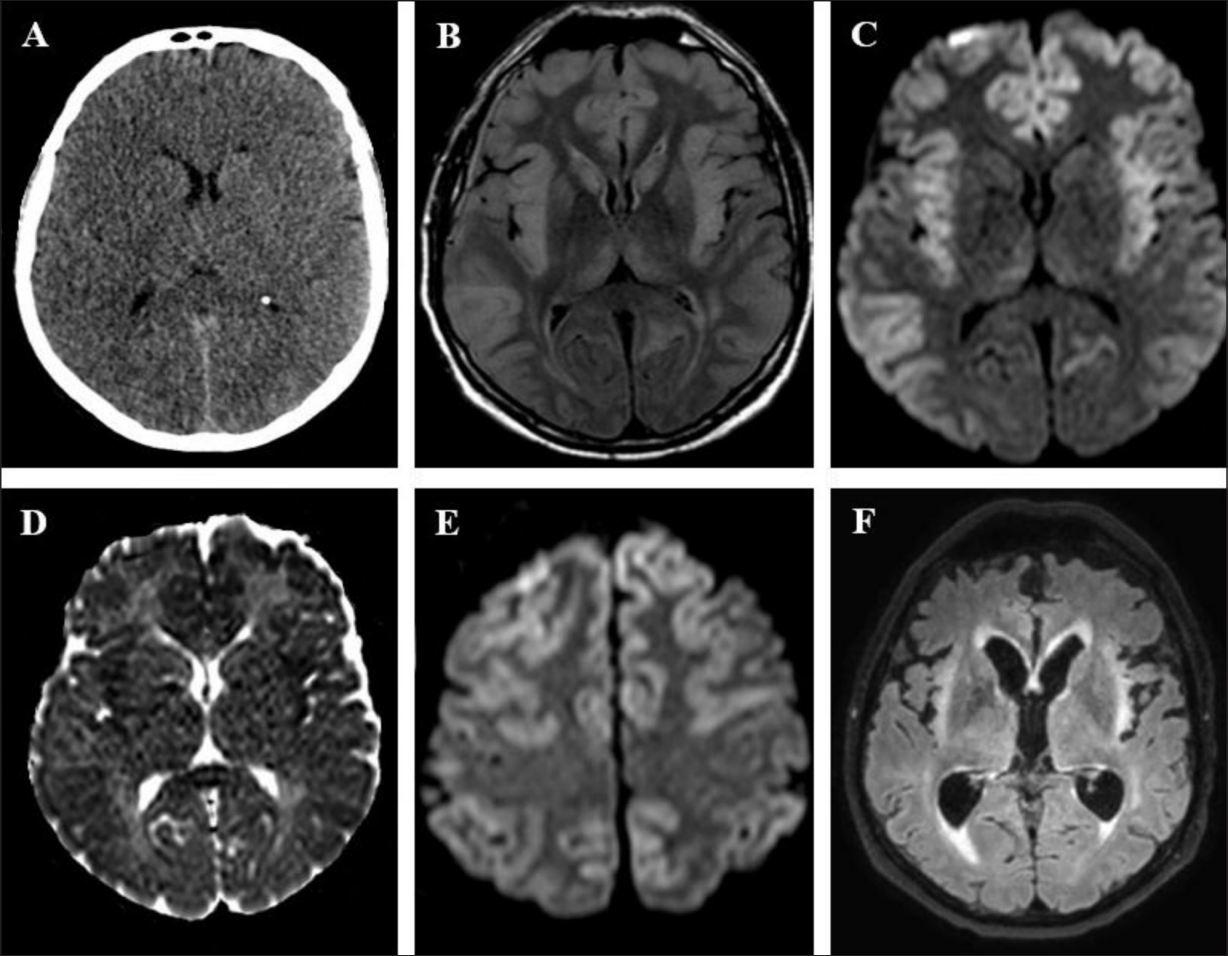
A 56-year-old man presented with status epilepticus and coma four days after bi-pulmonary graft. **(A)** Non-enhanced CT shows ventricular and sulcal effacement suggesting brain edema. **(B)** Fluid-attenuated inversion recovery (FLAIR) sequence shows bilateral cortical swelling and high signal, with sparing of the occipital cortex, as well as signal abnormalities in both caudate nuclei and thalami. **(C)** Diffusion weighted-imaging (DWI; b-value = 1000 s/mm^2^) and **(D)** apparent diffusion coefficient (ADC) map show respectively high and low signal of the cortex at the same levels, consistent with cytotoxic edema. **(E)** DWI sequence at a higher level shows sparing of the peri-rolandic cortex. **(F)** Follow-up FLAIR imaging at four weeks shows development of atrophy and gliosis in insula bilaterally.

A 56-year-old man developed a status epilepticus and coma four days after bipulmonary grafting for idiopathic pulmonary fibrosis. Neurological examination showed weakly reactive pupils. Liver and renal functions, glycemia and serum ion levels were normal. EEG displayed encephalopathy and epileptic activity. Serum ammonia was markedly increased at 661 μg/dl (normal value [NV]: 27–102 μg/dl). Bronchoalveolar microbial cultures indicated contamination of the pulmonary graft by *Ureaplasma urealyticum*, which was assumed to be the underlying cause of hyperammonemia. Figure 1 (B–E) shows the brain MRI findings nine days after onset of coma as well as the findings at follow-up (F) after four weeks. Despite aggressive therapy, the neurological status of the patient improved poorly.

### Case 2

**Figure 2:**
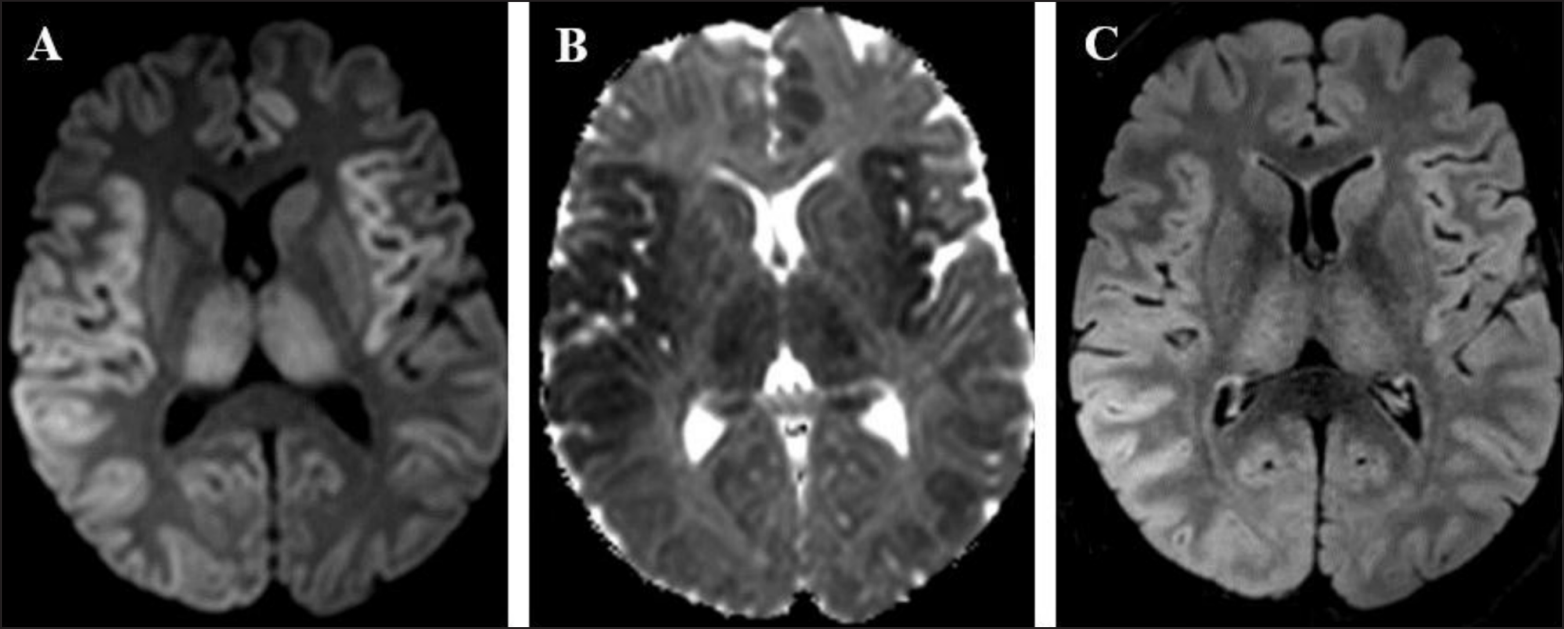
A 45-year-old man presented with seizures and fulminant hepatitis with hepatic failure, secondary to acetaminophen intoxication. **(A)** DWI (b-value = 1000 s/mm^2^) and **(B)** ADC map show cortical signal abnormalities in bilateral insulas and parietal lobes, left cingulate gyrus and both thalami, respectively high and low signal, consistent with cytotoxic edema. **(C)** FLAIR sequence shows high signal in the right parietal cortex and bilateral thalami.

A 45-year-old man with no known medical history was brought to the emergency department for convulsions with a GCS score of 5/15. Neurological examination showed anisocoria and pyramidal tract dysfunction. Blood tests were consistent with fulminant hepatitis complicated by hepatic failure, with serum ammonia >400 μg/dl. Serum acetaminophen level was 52.2 μg/ml (NV: 10–20). EEG showed diffuse severe encephalopathy. Figure 2 shows the brain MRI findings three days after admission. Despite treatment with N-acetylcysteine, the patient died of intracranial hypertension.

### Case 3

**Figure 3:**
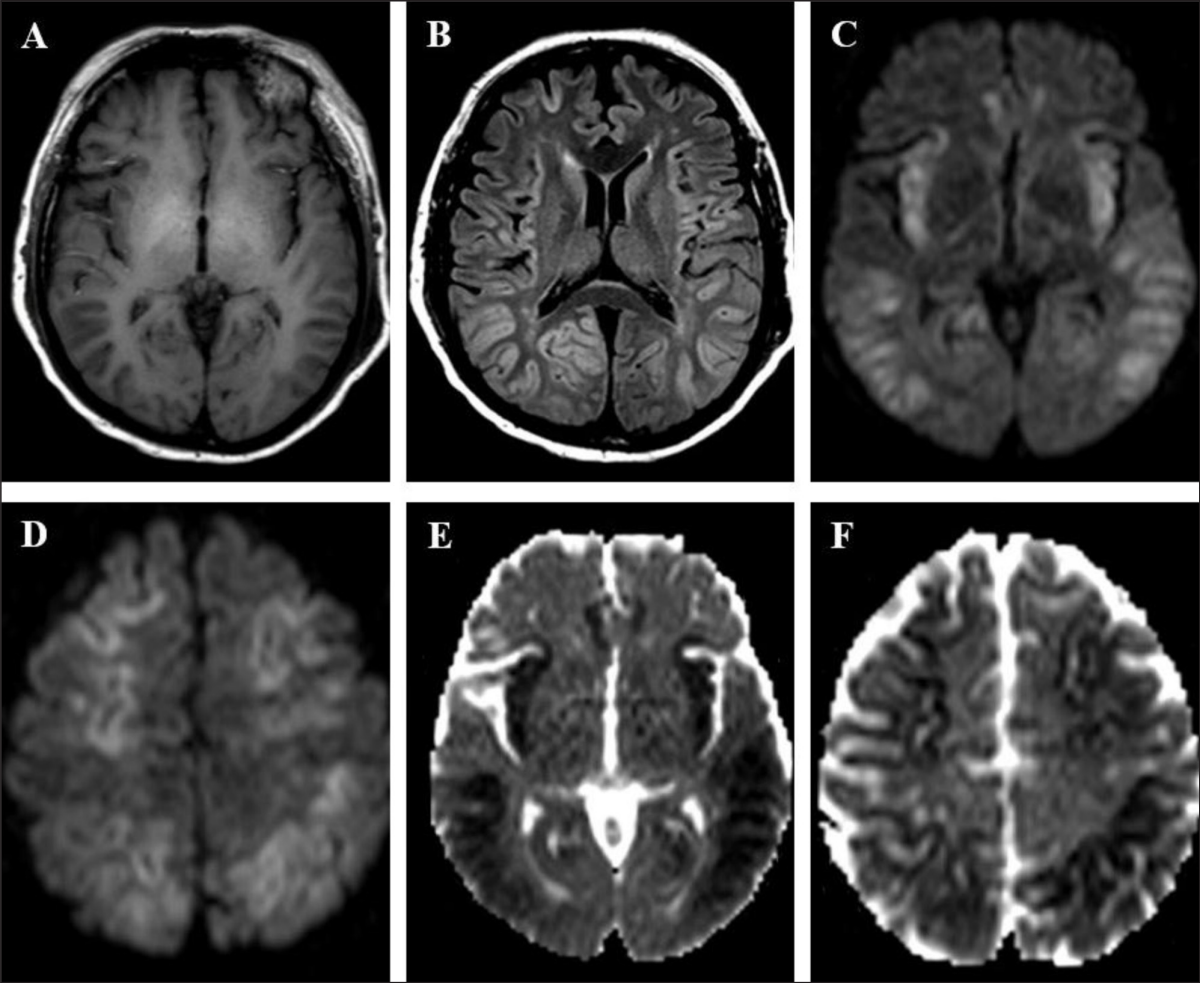
A 49-year old man was found in a coma. Clinical and laboratory findings indicated decompensated cirrhosis with encephalopathy. **(A)** T1-weighted imaging shows high signal in globi pallidi, suggesting underlying chronic hepatic disease. **(B)** FLAIR image shows bilateral and symmetric signal abnormalities in insular, cingulate, frontal and parietal cortices. **(C, D)** DWI (b-value = 1000 s/mm^2^) and **(E, F)** ADC show respectively high and low signal in affected cortices, consistent with cytotoxic edema, with sparing of occipital and peri-rolandic cortices.

A 49-year-old man was brought to the emergency department after being found unconscious. His medical history included alcoholism and chronic obstructive pulmonary disease. The GCS score was 3/15. Neurological examination showed horizontal eye deviation to the left and dilated but responsive pupils. Toxicologic screening was negative. Laboratory results were consistent with decompensated cirrhosis with serum level ammonia of 745 μg/dl. EEG showed epileptic activity with diffuse severe encephalopathy. A brain MRI (Figure 3) was performed three days after admission. Despite treatment, the patient died of septic shock due to pneumonia.

## Discussion

We reported three cases of acute hyperammonemic encephalopathy of different etiologies: infectious (case 1), toxic (case 2) and cirrhotic (case 3). In case 1, contamination of the pulmonary graft by *Ureaplasma* species was the cause of hyperammonemia, a known complication [2]. In case 2, the patient presented with fulminant acute hepatic failure following an overdose of acetaminophen. In case 3, hyperammonemia was related to decompensated cirrhosis.

Acute hyperammonemic encephalopathy might complicate acute or chronic liver disease and, less frequently, noncirrhotic conditions causing increased production or decreased elimination of ammonia, such as genetic disorders, drugs or bacterial infections [1]. In the pediatric population, it is most frequently the result of inborn errors of metabolism [3]. Acute hyperammonemic encephalopathy has various neuropsychiatric manifestations and can lead to coma and death, secondary to brain edema and intracranial hypertension [4]. The mechanism of cerebral toxicity is not yet elucidated. Physiologically, ammonia has various endogenous sources and is degraded in urea mostly by the liver. In hyperammonemia, the participation of brain in the metabolism of ammonia increases [5], ammonia crossing the blood-brain barrier by diffusion of its unionized form (NH_3_) and active transport of its ionized form (NH_4_+) [6]. In the astrocyte, glutamine synthetase metabolizes ammonia and glutamate into glutamine [7]. A major hypothesis is that glutamine, an osmolyte, causes water influx and swelling of the astrocytes, leading to brain edema [8]. Other hypotheses are that glutamine enters the astrocytic mitochondrion, initiating a reaction leading to cell death; or that glutamine may interfere with neurotransmission. Ammonia could also interfere with oxidative cerebral energy metabolism, inducing a high level of lactate [6].

The most striking MRI features described in acute hyperammonemic encephalopathy are bilateral symmetrical cortical signal abnormalities in the insula and cingulate gyrus [9,10,11,12,13,14,15], most often showing cytotoxic edema [10,12,13,14,15]. Involvement of frontal, temporal and parietal cortices, with various degrees of extension and symmetry, is almost always reported [10,11,12,13,14,15,16], along with sparing of occipital and peri-rolandic cortices [11,12,14,15]. Signal abnormalities in the thalami, once thought uncommon, are also a frequent finding [11,13,16]. Signal abnormalities have also been reported in the midbrain, peri-ventricular and sub-cortical white matter [9,16] but these seem to be less frequent. It is important to emphasize that lesion distribution seems to be independent of the origin of hyperammonemia.

In agreement with the literature, all three cases revealed cortical insular and cingulate cytotoxic edema – which was bilateral in cases 1 and 3 – while the occipital and peri-rolandic cortices were spared. Involvement of the thalami was present in cases 1 and 2. An unusual finding was involvement of the caudate nucleus in case 1: to the best of our knowledge, such a finding was described uniquely by Cifcti et al. [10] in a case of valproate toxicity. We observed no involvement of white matter. An additional finding was bilateral T1 hyperintensity in the globi pallidi in case 3, as may be seen in case of underlying hepatic chronic disease [16].

Extensive cortical signal abnormalities on brain MRI might be encountered in other conditions, namely hypoxic-ischemic encephalopathy, limbic encephalitis, acute hypertensive, hyponatraemic or Creutzfeldt-Jacob encephalopathies, that have therefore to be included in the differential diagnosis [17]. However, none of the patients had clinical or laboratory findings suggestive of any of the above-mentioned conditions and all of them could be easily ruled out. Seizures may also be associated with cortical abnormalities on peri-ictal MR imaging [18]. However, despite the presentation with seizures in cases 1 and 3, the observed MRI abnormalities, given their highly specific pattern, could be attributed to the main laboratory finding of hyperammonemia.

## Conclusion

The brain MRI findings in acute hyperammonemic encephalopathy are very specific. They typically include symmetrical signal abnormalities in insular and cingulate cortices with variable involvement of frontal, temporal, and parietal cortices, white matter, and thalami. Perirolandic and occipital cortices are usually spared. The radiologists should be able to recognize such findings in order to allow prompt treatment of the condition.
